# Electroencephalogram-Based Complexity Measures as Predictors of Post-operative Neurocognitive Dysfunction

**DOI:** 10.3389/fnsys.2021.718769

**Published:** 2021-11-10

**Authors:** Leah Acker, Christine Ha, Junhong Zhou, Brad Manor, Charles M. Giattino, Ken Roberts, Miles Berger, Mary Cooter Wright, Cathleen Colon-Emeric, Michael Devinney, Sandra Au, Marty G. Woldorff, Lewis A. Lipsitz, Heather E. Whitson

**Affiliations:** ^1^Department of Anesthesiology, Duke University School of Medicine, Durham, NC, United States; ^2^Duke Center for the Study of Aging and Human Development, Duke University School of Medicine, Durham, NC, United States; ^3^Hinda and Arthur Marcus Institute for Aging Research, Hebrew Senior Life and Harvard Medical School, Boston, MA, United States; ^4^Division of Gerontology, Department of Medicine, Beth Israel Deaconess Medical Center, Boston, MA, United States; ^5^Center for Cognitive Neuroscience, Duke University, Durham, NC, United States; ^6^Division of Geriatric Medicine, Department of Medicine, Duke University School of Medicine, Durham, NC, United States; ^7^Department of Psychiatry, Duke University, Durham, NC, United States; ^8^Department of Psychology and Neuroscience, Duke University, Durham, NC, United States; ^9^Geriatrics Research Education and Clinical Center, Durham VA Medical Center, Durham, NC, United States

**Keywords:** electroencephalogram (EEG), complexity, resilience, cognition, attention, anesthesia, delirium, perioperative medicine

## Abstract

Physiologic signals such as the electroencephalogram (EEG) demonstrate irregular behaviors due to the interaction of multiple control processes operating over different time scales. The complexity of this behavior can be quantified using multi-scale entropy (MSE). High physiologic complexity denotes health, and a loss of complexity can predict adverse outcomes. Since postoperative delirium is particularly hard to predict, we investigated whether the complexity of preoperative and intraoperative frontal EEG signals could predict postoperative delirium and its endophenotype, inattention. To calculate MSE, the sample entropy of EEG recordings was computed at different time scales, then plotted against scale; complexity is the total area under the curve. MSE of frontal EEG recordings was computed in 50 patients ≥ age 60 before and during surgery. Average MSE was higher intra-operatively than pre-operatively (*p* = 0.0003). However, intraoperative EEG MSE was lower than preoperative MSE at smaller scales, but higher at larger scales (interaction *p* < 0.001), creating a crossover point where, by definition, preoperative, and intraoperative MSE curves met. Overall, EEG complexity was not associated with delirium or attention. In 42/50 patients with single crossover points, the scale at which the intraoperative and preoperative entropy curves crossed showed an inverse relationship with delirium-severity score change (Spearman ρ = −0.31, *p* = 0.054). Thus, average EEG complexity increases intra-operatively in older adults, but is scale dependent. The scale at which preoperative and intraoperative complexity is equal (i.e., the crossover point) may predict delirium. Future studies should assess whether the crossover point represents changes in neural control mechanisms that predispose patients to postoperative delirium.

## Introduction

As the age of surgical patients has increased, efforts have focused on optimizing older adults’ postoperative brain health outcomes (Mahanna-Gabrielli et al., 2019) and avoiding perioperative neurocognitive disorders (PND; Evered et al., 2018) such as postoperative delirium. Up to half of older surgical patients experience some form of PND (Daiello et al., 2019). PND are distressing for patients and families and are associated with increased postoperative mortality (Loponen et al., 2008; Monk et al., 2008), long-term cognitive decline (Newman et al., 2001; Inouye et al., 2016), and increased long-term dementia risk (Lundstrom et al., 2003; Evered et al., 2016). Thus, there is urgent need for effective tools to identify those at highest risk for PND, so they can be targeted for delirium prevention interventions (Inouye et al., 2000; Chan et al., 2013; Ely, 2017).

Aside from delirium, the risk of other geriatric syndromes such as falls (Inouye et al., 2007) and frailty have been associated with age-related decreases in the physiologic output complexity of the associated organ systems (Costa et al., 2007; Manor et al., 2010; Wayne et al., 2014). Healthy organ systems are governed by networks of physiologic control mechanisms, which interact across multiple temporal and spatial scales to produce highly complex signals. The complexity of these physiological signals can be quantified using techniques adapted from information theory, such as multi-scale entropy (MSE; Costa et al., 2002). MSE works differently than traditional entropy measures such as sample entropy, which measure entropy over a single time scale and have maximal values with random noise. In contrast, MSE measures the likelihood that certain patterns of physiologic data are repeated in a time series at various levels of temporal resolution (i.e., duration). Specifically, MSE uses “coarse-graining” to transform the original temporal signal into multiple “scales” with increasingly lower temporal resolution. MSE then calculates the sample entropy of the coarse-grained signal at each scale. The more the repetition or regularity of the coarse-grained signal, the lower the sample entropy at its associated scale. Because MSE captures physiologic influences operating over different time scales at different frequencies, MSE separates “meaningful structural richness” (Costa et al., 2002) from noise in a way temporal, spectral, or traditional entropic analysis cannot. Increased signal complexity, quantified by the area under the MSE as a function of scale curve, has been associated with greater resilience –, i.e., greater capacity to adapt to stressors (Gijzel et al., 2019). This relationship between signal complexity and recovery from stressors is thought to reflect an organ system’s capacity to recover from perturbations, which depends on numerous interacting physiologic responses operating over varying time scales, with fluctuating patterns of recurrence. Further, signal complexity decreases with increasing age across many physiological systems. This age-related decline in complexity is associated with an impaired ability to recover from health stressors (Lipsitz, 2004; Zhou et al., 2017; Gijzel et al., 2019).

During the physiologic stress of surgery and anesthesia, frontal electroencephalogram (EEG) is widely used to titrate anesthetic administration (Berger et al., 2020; Chan et al., 2020). Intraoperative EEG has been studied to prevent delirium (and other forms of PND) in older adults (Chan et al., 2020), although protocols using traditional EEG metrics to titrate anesthetics have had inconsistent effects on PND rates (Chan et al., 2013; Radtke et al., 2013; Wildes et al., 2019; Evered et al., 2021). New complexity-based EEG analysis using MSE may predict delirium more accurately by accounting for complex physiological control mechanisms. Because anesthetics generally suppress excitatory neurotransmission (Franks and Lieb, 1994) and the complexity of neuronal functional connectivity decreases in sedated younger adults (Pappas et al., 2019), we hypothesized that complexity, and thus, the overall MSE of EEG signals (i.e., area under the MSE-by-scale curve) would decrease during general anesthesia.

In this work, we aimed to generate proof-of-concept data on the extent to which the complexity of peri-operative brain signals could identify patients likely to experience postoperative attentional deficits, including delirium. We focused on frontal EEG given the contribution of the frontal lobe to attention (Zanto and Gazzaley, 2019), the frontal location of common intraoperative EEG monitors (Chan et al., 2020), prior associations between frontal-EEG parameters and cognitive status (Giattino et al., 2017), and known anesthetic-induced age-related frontal-EEG changes (Purdon et al., 2013). Given this earlier work, we hypothesized that those with greater frontal-EEG complexity, either pre-operatively or intra-operatively, would have lower postoperative delirium incidence and severity and more resilient/better attention after surgery. We further hypothesized that a greater decrease in signal complexity from the pre-operative to intra-operative state might predict delirium.

## Methods

### Participants

This pilot study included 50 participants from the larger study, “Investigating Neuroinflammation Underlying Postoperative Brain Connectivity Changes, Postoperative Cognitive Dysfunction, Delirium in Older Adults” (INTUIT; Berger et al., 2019). The INTUIT study and this pilot analysis were approved by the Duke Health institutional review board. INTUIT is registered on clinicaltrials.gov clinical (NCT03273335). All participants, or legally authorized representatives, provided written informed consent prior to study participation.

INTUIT is an ongoing observational prospective cohort study on the role of neuroinflammation in post-operative cognitive dysfunction (POCD). INTUIT enrolls Duke patients age ≥ 60 years undergoing non-cardiac, non-neurological surgery (Berger et al., 2019). Exclusion criteria include age < 60 years, anticipated surgery duration < 2 h, incarceration, inadequate English fluency, and anticoagulant use that would preclude lumbar punctures. INTUIT has no cognitive exclusion criteria. In this pilot study, the first 53 INTUIT participants undergoing 32-channel EEG were considered. Two subjects were excluded due to insufficient post-operative delirium assessments. Another was excluded for insufficient usable intraoperative frontal EEG data, leaving *n* = 50.

### Delirium Severity and Attention Score

INTUIT participants undergo delirium screening prior to surgery (baseline) and twice daily after surgery while hospitalized with the 3-min confusion assessment method (3D-CAM; Marcantonio et al., 2014). Because more than a third of our participants were discharged by postoperative day 2, our analyses focused on 3D-CAM assessments from post-operative day 1. Delirium symptom severity score was calculated on our recently described 20-point scale (Vasunilashorn et al., 2020); higher scores indicate worse delirium signs and/or symptoms. We operationally defined an “attention score” as the number of correct answers on 3D-CAM items 4–7, which assess attention (Marcantonio et al., 2014), with higher scores therefore indicating better attention. The change in total 3D-CAM severity score and the change in attention score from before surgery to postoperative day 1 were calculated utilizing the first available 3D-CAM assessment on Day 1 (Berger et al., 2015a).

### Demographic and Clinical Variables

Demographic information (age, sex, race), baseline clinical status [depressive symptoms, cognitive performance, comorbidities, body-mass index (BMI), self-rated health status, and functional status], and type of surgery and anesthesia were obtained via survey administration or chart review as described (Berger et al., 2019).

### Electroencephalogram Recording and Processing

Thirty-two channel EEG recording was performed just before surgery for 3 min awake with eyes closed. The eyes-closed (rather than open) awake condition was chosen to exclude confounding by visual input in the awake state, since the eyes are typically closed during surgery. EEG was also obtained during anesthesia/surgery using the same 32-channel system.

For the first 10 subjects in this pilot study we used a tethered EEG cap (Woldorff et al., 2002) and recording system (BrainAmp MR Plus, Brain Products GmbH, Gilching, Germany). For subsequent subjects, a wireless recording system with a standard international 10–20 EEG cap configuration (LiveAmp, Brain Products GmbH, Morrisville, NC, United States) was used due to increased ease of use during surgery. Supplementary Figure 1A shows the electrode configurations of the two caps with the frontal region of interest, encompassing international 10–20 system sites Fp1, Fp2, F3, Fz, and F4, highlighted. EEG signals were recorded at a 1,000 Hz sample rate, with a 0.016–250 Hz passband, online Cz-electrode referencing, and electrode impedances < 20 kΩ. Surgical event markers (e.g., incision, electrocautery interference, end of surgery) were recorded and cross-referenced with the anesthetic record to ensure accuracy.

The methods of EEG post-processing and data selection have been described previously (Giattino et al., 2017) and are detailed in the Supplementary Material. 3-min segments of preoperative and intraoperative EEG data are selected according to specific criteria (see Supplementary Material/Methods) to ensure consistency and to make quasi real-time MSE calculations feasible in future applications. To remove as much mechanical/surgical artifact, electrical noise and electromyography (EMG) signal as possible, the raw EEG data were bandpass filtered using two hamming-windowed sinc FIR filters (high pass: 1 Hz half-amplitude cutoff with 1 Hz transition; low pass: 50 Hz half-amplitude cutoff with 20 Hz transition) before down-sampling to 250 Hz using EEGLAB (Delorme and Makeig, 2004).

### Multi-Scale Entropy Analyses

**FIGURE 1:**
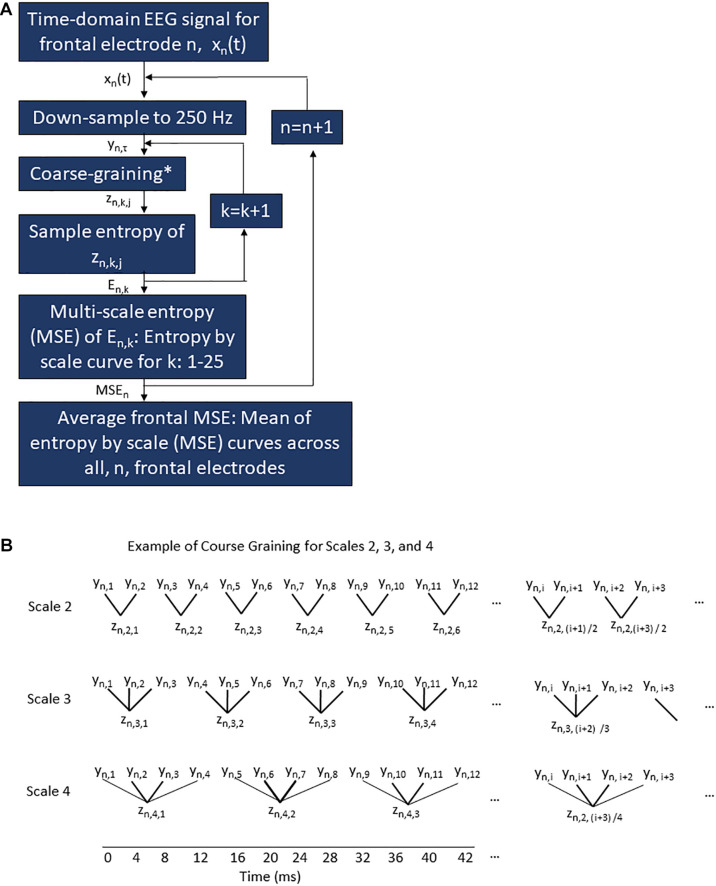
Multi-scale entropy procedure for frontal EEG electrodes **(A)** and example of course graining **(B)**. **(A)** A flow chart of the processing algorithm applied to the raw EEG signal for a given condition (pre-operative or intra-operative) for a given subject to generate the frontal average MSE curve for a given condition/subject. The time domain signal recorded at 1,000 Hz from a given frontal electrode, *n*, is represented as *x*_*n*_(*t*) where *t* ranges from 0 to 3 min. This raw signal *x*_*n*_(*t*) is down-sampled to 250 Hz to generate the time series *y*_*n*_,_τ_ where τ is the sample number from 1 to 45,000. Each sample encompasses 4 ms. The down-sampled time series *y*_*n*_,_τ_ is then course-grained for a given scale *k* (as shown in the examples of scale *k* = 2, 3, and 4 in **B**) to generate the coarse-grained signal *z*_*n,k,j*_, where *z*_*n,k* = 1,_*_*j*_* at scale 1 is identical to *y*_*n*,__τ_ and where *j* ranges from 1 to (τ + *k*–1)/*k*. The sample entropy is then calculated for each *z*_*n,k,j*_ signal to yield an entropy value *E*_*n,k*_. This is repeated for each scale *k*. Once *E*_*n,k*_ has been calculated for all scales *k* = 1–25, an MSE curve for a given subject, condition, and electrode, *n*. The MSE*_*n*_* curve ranges on the *x*-axis from scale *k* = 1–25. The MSE*_*n*_* curve on the *y*-axis is *E*_*n,k*_. This is repeated for every electrode, *n*, in the frontal region. Finally, the MSE curves for all *n* electrodes are averaged across scale *k* to yield a single frontal MSE curve for each subject/condition. **(B)** Example of course graining procedure perform on the down-sampled time series for electrode *n*, *y*_*n*,__τ_, where τ ranges from 1 to 4,500, to yield the course-grained series *z*_*n,k,j*_ where *n* represents the electrode in question, *k* is the scale value, and *j* is the sample number. Scales 2, 3, and 4 are shown for examples above, but this same pattern continues up to scale 25. The temporal resolution associated with the course graining is show on the axis at the bottom of the figure.

Multi-scale entropy analysis was performed on the frontal EEG data from each participant’s 3-min awake eyes-closed and 3-min intraoperative segments by researchers blinded to delirium outcomes. MSE analysis, which is depicted in Figure 1A, calculates the sample entropy of the post-processed signal from each electrode down-sampled to 250 Hz (scale 1) and of coarse-grained signals (scales 2–25). Coarse graining averages sequential data points to create a new coarse-grained signal at each scale (Figure 1B), as originally described by Costa et al. (2002). For example, at the scale of 2, pairs of non-overlapping sequential data samples are averaged to generate a new temporal signal with half as many samples as the original (i.e., 8 ms/sample at scale 2, compared with 4 ms/sample in the original signal at scale 1). For the scale of 3, sequential non-overlapping triplets of data were averaged to generate a signal with a third as many samples as the original (i.e., 12 ms/sample). The relationship between sample length, *L*, of the coarse-grained signal, *l* the sample length of the original signal (i.e., 4 ms) and scale, *s*, is shown in Eq. 1.

(1) L=s×l

Coarse graining was performed for scales up to 25 (i.e., 100 ms/sample). The sample entropy of each of these coarse-grained signals was calculated to yield MSE values at scales 2, 3, …, 25 (Costa et al., 2002). Plotting entropy values for scale yielded a discrete entropy-by-scale (MSE) curve from scales 1 to 25. Finally, for each subject and condition, the individual MSE curves for each electrode within the frontal region were averaged to give a mean frontal MSE curve. An effect of coarse graining is removal of higher frequency signal information by averaging successive points together. By the Nyquist theorem, only frequencies lower than 1/ (2 × *L*_*s*_), where *L*_*s*_ is the sample length of the coarse-grained signal at scale *s* from Eq. 1, can be fully represented in the coarse-grained signal.

### Electroencephalogram Predictor Variables Derived From Multi-Scale Entropy Curves

Area under the curve (AUC) of the entropy-by-scale curves for each condition was calculated with a trapezoidal approximation as shown in Eq. 2, where *y* is the MSE value at a given scale and *n* is 25.

(2) $$\text{AUC}=0.5\times y_{1}+\sum_{i=2}^{n-1}y_{i}+0.5\times y_{n}$$

Predictor variables for each participant included MSE curve AUC for the pre-operative eyes-closed and for the intra-operative conditions, and the difference in AUC between these conditions.

### Statistical Analysis

All statistical analyses were performed by a statistician who did not collect data or process EEG data. First, we compared the AUC of the MSE curves in the pre-operative and intra-operative conditions, to determine whether average complexity across time scales increased or decreased significantly between these two conditions. For each participant, we calculated the difference between AUC values in the pre-operative and intra-operative condition, and we used *t*-tests to determine whether these values differed significantly from zero.

Next, we examined the distribution of demographic and clinical variables in our cohort. To identify variables that might have confounded the relationship between EEG metrics and the neurocognitive outcomes of interest, we split the cohort into participants who did or did not exhibit a decrease in attention score after surgery. We compared the characteristics of these sub-groups with *t*-tests for continuous variables or chi-squared, Wilcoxon rank sum, or Fisher tests for proportional variables.

Finally, we modeled the relationship between each EEG predictor variable and two dependent variables, namely the change from baseline to postoperative day 1 in delirium-symptom severity and in attention scores. Initially, we calculated Spearman’s correlation coefficients to estimate relationships between variables of interest. If an association was detected with a ρ value of 0.25 or greater, we further modeled the relationship. We used linear regression to model the change in delirium symptom scores, and proportional-odds analysis to model the change in attention scores due to the small range in scores. In adjusted models, we included covariates that differed significantly between individuals with versus without an attention-score decrease.

## Results

### Participant Characteristics, in Relation to Neurocognitive Outcomes

Baseline subject characteristics are shown in Table 1, surgical, anesthetic, and pharamacological factors are shown in Supplementary Table 1. Almost all participants underwent general anesthesia (*n* = 47). The rest (*n* = 3) received propofol sedation and regional or neuraxial anesthesia without intubation; none of these participants experienced delirium or attention score decreases on post-operative day 1.

**TABLE 1:** Pre-operative characteristics of all participants and a comparison of participants with and without a decrease in attention score on Day 1 after surgery.

**Variable**	**Overall cohort (*N* = 50)**	**No decrease in attention score (*N* = 32)**	**Decrease in attention score (*N* = 18)**	***p*-value**

**Demographics**				
Age (years), mean (SD)	68.8 (5.4)	67.7 (4.4)	70.7 (6.5)	**0.05^1^**
Race/Ethnicity, *n* (%) White Black Other	40 (80.0%) 9 (18.0%) 1 (2.0%)	27 (84.4%) 5 (15.6%) 0 (0.0%)	13 (72.2%) 4 (22.2%) 1 (5.6%)	0.32^2^
Female gender, *n* (%)	25 (50.0%)	18 (56.3%)	7 (38.9%)	0.24^2^

**Psychosocial characteristics**				

Years of education, median [Q1, Q3]	16 [14,17]	16 [14,17]	16 [14,17]	0.97^3^
MMSE score, median [Q1, Q3]	28 [26, 29]	28.5 [27, 29]	27.5 [24, 29]	0.32^3^
Cognitive impairment (MMSE < 26), *n* (%)	10 (20.0%)	4 (12.5%)	6 (33.3%)	0.14^4^
Baseline attention score, *n* (%) 1 2 3 4	1 (2.0%) 4 (8.0%) 11 (22.0%) 34 (68.0%)	0 (0.0%) 3 (9.4%) 9 (28.1%) 20 (62.5%)	1 (5.6%) 1 (5.6%) 2 (11.1%) 14 (77.8%)	0.30^4^
CES-depression, median [Q1, Q3]	7 [4, 16]	7 [5, 15]	8 [4, 16]	0.98^3^

**Pre-surgical health status**				

Self-rated health, *n* (%) Excellent Very good Good Fair Poor	8 (16.0%) 19 (38.0%) 17 (34.0%) 6 (12.0%) 0 (0.0%)	6 (18.8%) 13 (40.6%) 10 (31.3%) 3 (9.4%) 0 (0.0%)	2 (11.1%) 6 (33.3%) 7 (38.9%) 3 (16.7%) 0 (0.0%)	0.73^2^
IADL score, median [Q1, Q3]	6 [6, 6]	6 [6, 6]	6 [6, 7]	0.41^3^
Body mass index (kg/m^2^), mean (SD)	29.2 (5.6)	30.6 (5.8)	26.8 (4.3)	0.02^1^

**Comorbidities**				

Diabetes, *n* (%)	6 (12.0%)	3 (9.4%)	3 (16.7%)	0.65^4^
Chronic lung disease, *n* (%)	8 (16.0%)	5 (15.6%)	3 (16.7%)	>0.99^4^
Cardiovascular disease, *n* (%)	12 (24.0%)	4 (12.5%)	8 (44.4%)	**0.02^4^**
Renal disease (mod-severe), *n* (%)	5 (10.0%)	2 (6.3%)	3 (16.7%)	0.34^4^
Cerebrovascular disease, *n* (%)	1 (2.0%)	0 (0.0%)	1 (5.6%)	0.36^4^
Thyroid disease, *n* (%)	10 (20.0%)	8 (25.0%)	2 (11.1%)	0.30^4^
Rheumatoid arthritis, *n* (%)	1 (2.0%)	1 (3.1%)	0 (0.0%)	>0.99^4^

*P-value key: 1 = *t*-test, 2 = Chi-Square, 3 = Wilcoxon rank sum, 4 = Fisher.*

*MMSE, mini mental status exam; IADL, instrumental activities of daily living; and MAC, monitored anesthesia care.*

*Values that are significant or nearly significant are bolded.*

While only seven members of this cohort (14%) screened positive for delirium on post-operative day 1, 18 (36%) had a worse 3D-CAM attention score after versus before surgery. Compared to participants with stable-to-improved attention scores on post-operative day 1, participants whose attention scores decreased were more likely to have lower BMI, more cardiovascular disease, and older age (Table 1). None of the other clinical or demographic variables in Table 1 or Supplementary Table 1 differed significantly between those with versus without a worsened attention score on postoperative day 1.

### Electroencephalogram Complexity in the Pre-operative and Intra-operative Conditions

**FIGURE 2:**
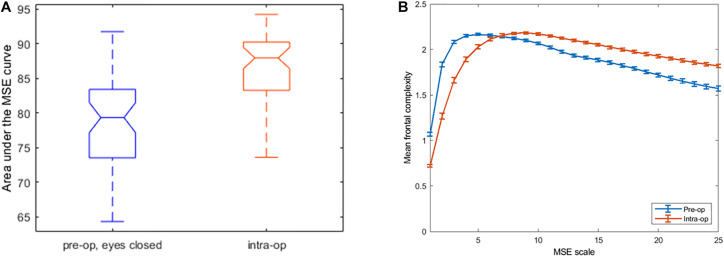
AUC whisker plots **(A)** and entropy-by-scale curves **(B)**, averaged across 50 participants. **(A)** Area under the curve (AUC) for the frontal entropy-by-scale curves for the pre-op eyes-closed awake condition and the surgical/anesthetized condition. AUC complexity values integrated across all 25 scales are higher during anesthesia/surgery relative to the conscious awake condition. **(B)** Frontal complexity (entropy) values (*Y* axis), by time scale (*X* axis), averaged across all participants with standard error bars shown. The blue line represents the preoperative awake/eyes closed EEG recording. The red line represents intraoperative/anesthetized EEG recording. Note the clear crossover point between scales 7 and 8, where the pre-operative complexity values are higher (compared to intra-operative complexity values) to the left of this point but are lower to the right of this point.

The average frontal EEG complexity, measured as the area under the entropy-by-scale curve across all 25 scales, was greater in the intra-operative, anesthetized condition than in the pre-operative, eyes closed condition (*p* = 0.0003; Table 2 and Figure 2A). The mean difference between the AUCs was 1.6 units (standard deviation 2.9 units); overall, this result was opposite to our original hypothesis.

**TABLE 2:** Summary of EEG multi-scale entropy area under the curve (AUC) in pre-operative (awake, eyes closed) and intra-operative conditions (*n* = 50).

	**Mean (SD)**	**Median [Q1, Q3]**	**Min, max**
Pre-operative AUC	45.19 (2.25)	45.39 [43.83, 46.87]	39.06, 49.14
Intra-operative AUC	48.82 (1.43)	47.32 [46.33, 47.84]	43.03, 48.95
Difference in AUC in pre-operative versus intra-operative condition	1.62 (2.92)*	1.52 [−0.17, 3.11]	−5.83, 9.48

*t-test (for difference from 0), *p* = 0.0003.

#### Multi-Scale Entropy Curve “Crossover” Phenomenon

While plotting MSE as a function of scale, we observed that subjects’ pre-operative eyes-closed and intra-operative mean MSE curves typically intersected or “crossed over” each other, with greater complexity values at longer scales during anesthesia/surgery than before, and the converse at shorter scales, as shown in the across-subject grand averages in Figure 2B. A 2 × 25 repeated-measures ANOVA (2 conditions, 25 scales) applied to all 50 subjects confirmed this relationship, reflected by a highly significant interaction of pre- versus intra-operative condition × scale (*p* < 0.001). Most subjects, 84% (42 of 50), had a single “crossover” point, but the eight subjects with multiple “crossovers” did not differ clinically or demographically from the rest of the cohort, as shown in Supplementary Table 2.

Upon identifying this MSE-by-scale “crossover” phenomenon, we wanted to examine its intra-subject consistency and its relationship to delirium and inattention outcomes. In particular, we wanted to analyze the information provided by the scale at which the crossover occurred (i.e., the crossover point). To take the more conservative approach, we applied these analyses to those subjects (42 of 50) who had only one MSE curve intersection in the 25 scales analyzed. For these subjects, the scale of the “crossover” points ranged from 3 to 15 with a median [Q1, Q3] of 7 [6,10] and a mode of 7 (12/42). The “crossover” points were nearly normally distributed with a slight rightward skew (Shapiro–Wilks test, *p* = 0.015).

To assess the intra-subject consistency of the crossover-point across recording time, we split the pre-operative eyes-closed and intra-operative EEG recordings into equal sub-segments and calculated the “crossover” point for the four permutations of these sub-epochs (i.e., first half pre-op v first half intra-op, first half pre-op v. second half intra-op, second half pre-op v. first half intra-op, and second half pre-op v. second half intra-op). The four permutated crossover points agreed very closely with the original crossover points obtained from the full segments, as shown in Supplementary Figure 1B. Further, a Friedman test of the four permutations by subject showed no statistically significant differences in “crossover” point as a function of sub-epochs (*Q* = 1.59, *p* = 0.66).

### Relationship Between Electroencephalogram Complexity Metrics and Post-operative Cognitive Scores

We hypothesized that the “crossover point” of the multiple scale entropy as a function of scale might capture clinically useful information about the impact of surgery and anesthesia on brain activity. Thus, we evaluated complexity-based EEG metrics (AUC values, “crossover” point) as potential predictors of the change in delirium-symptom severity or attention scores from baseline to postoperative day 1 (Table 3). Correlations between AUC values and these cognitive outcomes were weak (absolute value ρ < 0.15 for all correlations, *p* > 0.05 for all). However, among the 42 participants whose MSE curves crossed only once, there was a potential association between the “crossover” point occurring at a longer scale and more favorable cognitive outcome. The correlation between the “crossover” point and the change in attention score came very close to reaching statistical significance (ρ = 0.29, 95% confidence interval [CI] -0.02 to 0.55, *p* = 0.060). In a regression model that adjusted for the three clinical variables found to differ with decreased post-operative attention score (i.e., lower BMI, cardiovascular disease, and increased age), a similar, nearly significant relationship was observed between crossover point and change in attention score (proportional odds ratio 1.25, 95% CI 0.99–1.59, *p* = 0.07). The “crossover” point similarly showed a potential correlation with pre- to post-operative delirium severity score change (ρ = −0.31, 95% CI −0.57 to 0.01, *p* = 0.054).

**TABLE 3:** Association between EEG predictor variables and cognitive score changes from baseline to post-operative day 1.

	***Cognitive outcome variable: Change in attention score***					
**EEG predictor variable**	**Correlation**		**Unadjusted proportional odds models**		Adjusted proportional odds models*	
	**Spearman ρ (95% CI)**	***p*-value**	**Odds ratio (95% CI)**	***p*-value**	**Odds ratio (95% CI)**	***p*-value**
Pre-operative AUC	0.10 (−0.18, 0.37)	0.49				
Intra-operative AUC	−0.05 (−0.32, 0.23)	0.73				
Difference in AUC	−0.12 (−0.39, 0.16)	0.39				
Crossover location (*N* = 42**)	0.29 (−0.02, 0.55)	0.060	1.25 (0.99, 1.58)	0.058	1.25 (0.99, 1.59)	0.066

	***Cognitive outcome variable: Change in delirium severity score***					
**EEG predictor variable**	**Correlation**		**Unadjusted linear regression models**		Adjusted linear regression models*	
	**Spearman ρ (95% CI)**	***p*-value**	**Mean difference (95% CI)**	***p*-value**	**Mean difference (95% CI)**	***p*-value**

Pre-operative AUC	−0.08 (−0.35, 0.22)	0.61				
Intra-operative AUC	0.06 (−0.23, 0.34)	0.67				
Difference in AUC	0.17 (−0.12, 0.44)	0.24				
Crossover point (*N* = 42**)	−0.31 (−0.57, 0.01)	**0.054**	−0.10 (−0.23, 0.03)	0.11	−0.10 (−0.23, 0.03)	0.12

*Adjusted proportional odds model included the following covariables: age, body mass index, cardiovascular disease.

**Restricted analysis excludes eight participants with atypical MSE curves (multiple crossover points).

*Values that are significant or nearly significant are bolded.*

## Discussion

To our knowledge, this is the first study to examine the extent to which EEG signal complexity in the awake condition versus the anesthetized/surgical condition is associated with postoperative delirium or inattention severity. To quantify EEG complexity, we calculated entropy at MSE and observed several key patterns. First, the total complexity across all times scales (AUC of the MSE values) was significantly greater in the anesthetized than in the awake eyes-closed condition, opposite from our *a priori* hypothesis. These results are consistent with previous studies (Wang et al., 2014; Eagleman et al., 2018) that reported increased entropy during deep anesthesia compared with the awake eyes-closed condition, although that study used a different summating entropy measure and was applied only across the longer temporal scales that were analyzed here. Importantly, however, in the current study we also observed a highly significant and robust interaction of pre- versus intra-operative condition and temporal scale, with the MSE values at the shorter time scales being higher during the awake than during the anesthetized condition, but with the converse relationship at longer time scales. The change seen within individual subjects during anesthesia compared to the awake state, mirrors the differences reported between health controls and awake older adults with Alzheimer’s disease (Mizuno et al., 2010). Specifically, EEG complexity determined via MSE was lower in AD patients relative to healthy controls at short time scales, possibly reflecting less regular structured neuronal circuit activity in AD (or, in this study, during anesthesia and surgery). In AD patients, EEG MSE was higher at longer-time scales, possibly reflecting a more “disconnected” brain in which AD “brain activity [tending more] toward random or non-stationary” neuronal activity (Yang et al., 2013). It is certainly possible that during anesthesia/surgery, “regular” neuronal circuit patterns are lower (evidenced by lower shorter scale MSE) and overall brain electrical activity is more “random” (evidenced by higher longer scale MSE), leading to the observed crossover point. The scale at which this “crossover” phenomenon occurred varied amongst individuals but appeared to be highly consistent within subject.

Our data are unique in showing that the complexity of a physiologic signal may change in different ways during an acute stressor depending on the scale at which it is measured. This certainly makes sense if different scales result from the outputs of multiple physiologic control mechanisms, each acting over its own frequencies and timeframe, yet all dynamically and adaptively responding to the same physiological stressors, namely anesthesia and surgery. By examining entropy values across a wide range of temporal scales, we show a robust, highly significant, crossover pattern of the MSE-by-scale curves. In addition, we describe the potential clinical significance of this crossover phenomenon. More specifically, participants whose MSE-curve crossover point occurred at shorter versus longer temporal scales tended toward greater delirium symptomatology on the first day after surgery.

While this relationship did not quite reach statistical significance in this small pilot study, such a result may reflect a lack of sufficient statistical power (a type II statistical error). It is also worth noting that delirium typically presents on postoperative day two (Robinson et al., 2009), a day later than was feasible to assess in this pilot cohort, which may have reduced our ability to detect a statistically significant relationship between crossover point and delirium occurrence.

Nonetheless, if future studies demonstrate a significant association between the crossover point and postoperative delirium or inattention risk, then such a measure could potentially be used clinically (e.g., built into perioperative EEG monitors) to identify target patients for scarce delirium prevention resources such as the HELP program (Inouye et al., 2000; Ely, 2017) or ABCDEF bundle (Ely, 2017). The widespread use of intraoperative frontal EEG monitoring suggests that this would be feasible. Further, if future studies confirm the intra-subject crossover point stability observed here, then the crossover point could simply be calculated once per surgery/anesthetic, allowing clinicians to arrange postoperative delirium prevention interventions before the surgery/anesthetic is completed.

In addition to the clinical-relevance of frontal EEG monitoring, our proof-of-concept analysis focused on the frontal EEG because of its previous associations with cognitive processes (Babiloni et al., 2016; Giattino et al., 2017; Koch et al., 2019). We accounted for advances in clinical anesthesiology practice by selecting and analyzing EEG epochs that were free of artifacts, adjunct anesthetic drug exposure, and burst suppression. Intraoperative burst suppression has been associated with postoperative delirium in prior studies (Soehle et al., 2015; Kratzer et al., 2020; Pedemonte et al., 2020; Shao et al., 2020), yet modest reductions in burst suppression have not been shown to reduce postoperative delirium incidence (Wildes et al., 2019; Tang et al., 2020). Our data raise the possibility that EEG metrics that are present even in the absence of burst suppression may be associated with post-operative neurocognitive outcomes, similar to our prior finding that lower intraoperative alpha power is associated with lower preoperative cognitive function (Giattino et al., 2017).

While most prior research on EEG as a predictor of delirium has predominantly relied on analysis of EEG data collected either intra-operatively (Kratzer et al., 2020; Sun et al., 2020) or pre-operatively (van Montfort et al., 2020), here we compared the pre-operative and intra-operative EEG signals. We observed relative changes in EEG complexity from before to during anesthesia/surgery, which may reflect brain-health vulnerability. While we did not find an association between dose or administration of common anesthetics, analgesics, or neuromuscular blockers and cognitive outcomes (Supplementary Table 1), the overall stress of surgery/anesthesia may exacerbate pre-existing neurocognitive vulnerability in older adults. In other physiologically stressful situations (e.g., walking on uneven ground), age-related decreases in the complexity of associated physiological variables (e.g., postural sway) predict adverse outcomes (e.g., falls); presumably, decreased physiologic complexity reflects diminished inherent capacity to respond adaptively to stress (Lipsitz, 2004; Manor et al., 2010; Wayne et al., 2014; Zhou et al., 2017). Similarly, EEG signal changes in response to surgery/anesthesia may be an indicator of the brain’s ability to function normally during and following these stressors.

The relationship between EEG complexity (i.e., MSE) and more traditional EEG power by frequency analysis merits further study. EEG frequency bands do not map linearly to MSE scales although higher frequency information corresponds to shorter scales, while lower frequency information corresponds to longer scales (Takahashi et al., 2009). Thus, while higher intraoperative MSE values at longer scales may roughly reflect the tendency toward higher frontal power in lower EEG frequency bands (e.g., delta, alpha) during anesthesia (Purdon et al., 2013, 2015), greater MSE values reflect higher *complexity/entropy*, not necessarily higher power (i.e., a large but very regular low-frequency wave could have high power but rather low complexity). Thus, these MSE patterns may reflect important additional neurophysiologic processes not measured by traditional EEG power measures.

Beyond applying entropy-based analysis to both preoperative and intraoperative EEG, we characterized the “crossover” phenomenon and evaluated its relationship with delirium and inattention. Subjects whose MSE curves intersected at short scales, i.e., with fewer scales at which intraoperative entropy was lower than pre-operative entropy, tended to have worse delirium/inattention although these results narrowly missed statistical significance at α = 0.05. A few participants had multiple MSE “crossovers.” While our data do not provide an obvious explanation for how these individuals differed from the rest, the presence of multiple crossover points literally means that there were multiple temporal scales in which that patient’s EEG complexity was the same in the awake and anesthetized states. Those who had multiple “crossover” points tended to undergo sedation without intubation/general anesthesia (2 of 8 subjects, compared to no subjects in the single crossover group) or to have undergone orthopedic surgery (3/8 of the multiple crossover group compared with 6/42 in the single crossover group). We hypothesize that those with multiple crossover points have more similar neurophysiological complexity in the anesthetized and awake state, which could occur if the patient was lightly anesthetized. This should be explored in future work with more homogeneity in anesthetic and surgical exposures.

This study has several limitations. First, we enrolled a small sample at a single center. Due to its exploratory nature, the study was not powered *a priori* to detect any particular entropic change, much less the “crossover” phenomenon that became apparent during our analysis. Second, as previously mentioned, our patients’ short post-surgical hospitalization only allowed for post-operative day 1 3D-CAM scores. Though consistent across our study population, the post-operative day 1 assessment may have missed later post-operative delirium or inattention, which most frequently develops on postoperative day 2 (Robinson et al., 2009). Third, the heterogeneous types of surgery and anesthesia studied here complicate interpretations about potential mechanism(s) underlying different EEG patterns and could potentially confound the relationship between these EEG patterns and postoperative delirium/inattention. Fourth, this study focuses specifically on post-operative delirium; however, POCD more broadly defined (Berger et al., 2015b) is another important perioperative cognitive outcome that could be studied in future work. Finally, despite bandpass filtering 1–50 Hz before down-sampling the EEG during preprocessing (which removes all frequencies greater than 50 Hz), we cannot rule out the possibility that some EMG signal remains with the EEG data. The possibility of EMG contamination is a problem for EEG studies in general, and larger studies could partly address this by controlling for depth of paralysis. Nonetheless, the significant and nearly significant findings in our work would have been unlikely to be attributable to EMG alone.

These limitations notwithstanding, this is the first study to describe this EEG MSE crossover phenomenon and its potential association with delirium. Further, we demonstrate the potential benefit of comparing the pre-operative and intra-operative EEG signals as they may contain clinically useful information on a condition by scale basis. If future research finds that the “crossover” point is predictive of delirium, studying its mechanistic basis may shed light on the etiology of delirium itself.

## Data Availability Statement

The datasets presented in this article are not readily available because data is part of a larger clinical trial that is on-going and therefore not yet available for public use. Requests to access the datasets should be directed to MB, miles.berger@duke.edu.

## Ethics Statement

The studies involving human participants were reviewed and approved by Duke Health Institutional Review Board. The patients/participants provided their written informed consent to participate in this study.

## Author Contributions

LA, MB, MGW, and HW wrote the document. HW, CC-E, and MB were principal investigators of funded projects that supported this work. MB, CH, SA, CG, and KR were involved in participant recruitment and data acquisition from human subjects. LL and JZ provided expertise and entropic analysis with BM. LA and KR performed the EEG analysis under the guidance of MGW. KR and CG performed the EEG preprocessing and segment selection under the guidance of MGW. MCW performed the statistical analysis. MD and CC-E offered feedback on the manuscript to refine the finished product. All authors contributed to the article and approved the submitted version.

## Conflict of Interest

The authors declare that the research was conducted in the absence of any commercial or financial relationships that could be construed as a potential conflict of interest.

## Publisher’s Note

All claims expressed in this article are solely those of the authors and do not necessarily represent those of their affiliated organizations, or those of the publisher, the editors and the reviewers. Any product that may be evaluated in this article, or claim that may be made by its manufacturer, is not guaranteed or endorsed by the publisher.

## Supplementary Material

Supplementary Figure 1 **(A)** Electrode montages for the two EEG cap systems used. *Tethered custom system (left). Wireless system (right). The frontal region of interest (ROI) for each is highlighted in Red on both.* Frontal channels include Fp1, Fp2, 9, 10, 11, and 12 in the custom cap (right) and Fp1, Fp2, F3, F4, and Fz with the standard 10–20 configuration (left). **(B)** Mean (left) and median (right) differences in scale value across 4 permutations of sub-epochs and the original crossover point for each subject calculated from the full epoch. All subjects had mean and median differences of 1 scale value or less.

Supplementary Table 1 Intraoperative characteristics of all participants and comparison of participants with and without a decrease in attention score on Day 1 after surgery. aaMAC, age-adjusted minimum alveolar concentration.

Supplementary Table 2 Comparison of participants with and without a crossover point in their pre-operative and intra-operative MSE curves.
