# Supplementary figures and images for: Electroencephalogram-Based Complexity Measures as Predictors of Post-operative Neurocognitive Dysfunction
Source: Front Syst Neurosci. 2021 Nov 10;15:718769. doi: 10.3389/fnsys.2021.718769 (PMC8631543; doi:10.3389/fnsys.2021.718769)

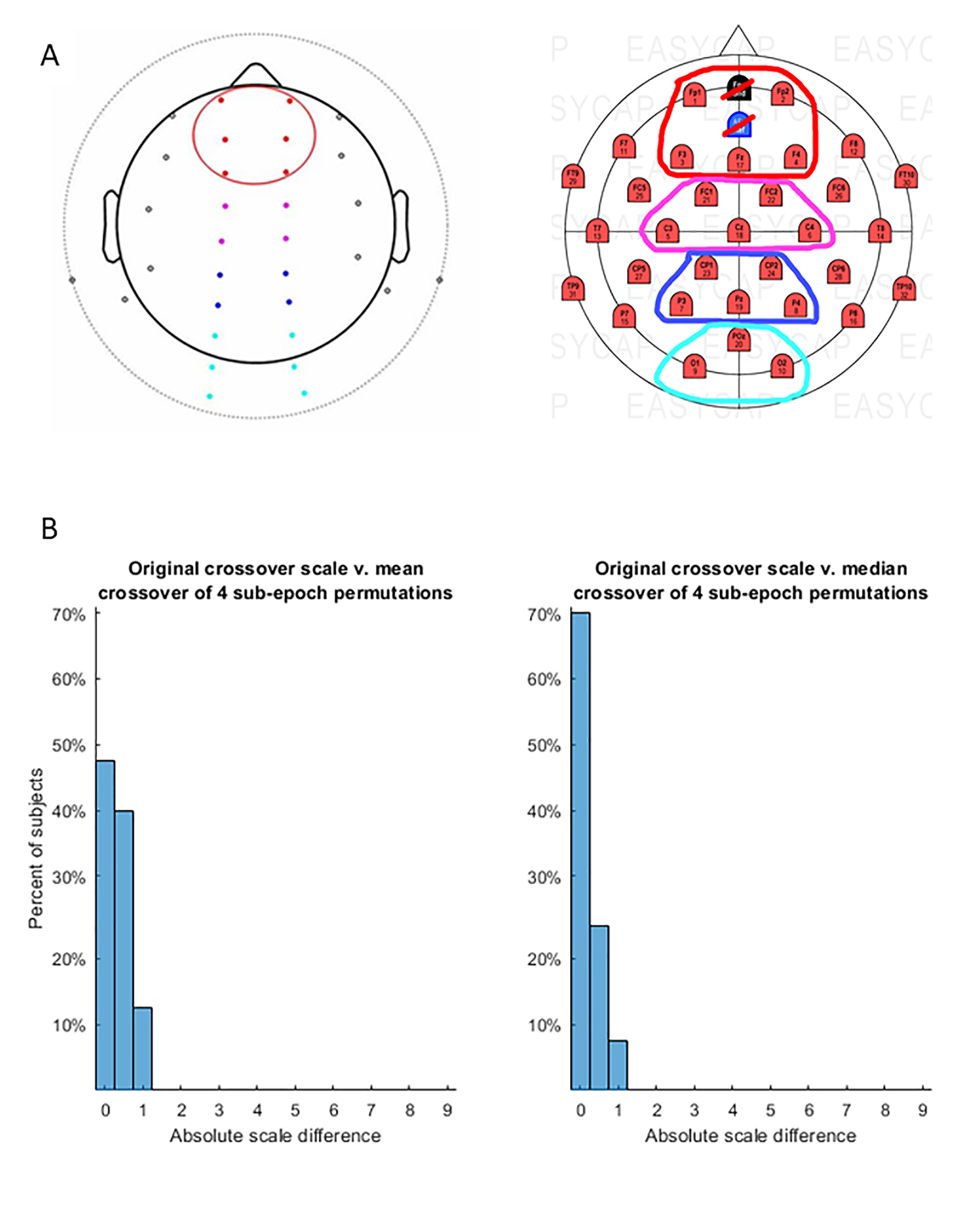

Supplement: Supplementary Figure 1 — (A) Electrode montages for the two EEG cap systems used. Tethered custom system (left). Wireless system (right). The frontal region of interest (ROI) for each is highlighted in Red on both. Frontal channels include Fp1, Fp2, 9, 10, 11, and 12 in the custom cap (right) and Fp1, Fp2, F3, F4, and Fz with the standard 10–20 configuration (left). (B) Mean (left) and median (right) differences in scale value across 4 permutations of sub-epochs and the original crossover point for each subject calculated from the full epoch. All subjects had mean and median differences of 1 scale value or less. [file Image_1.TIF]
