# Supplementary material for: Electroencephalogram-Based Complexity Measures as Predictors of Post-operative Neurocognitive Dysfunction
Source: Front Syst Neurosci. 2021 Nov 10;15:718769. doi: 10.3389/fnsys.2021.718769 (PMC8631543; doi:10.3389/fnsys.2021.718769)
Supplement: Supplementary Table 1 — Intraoperative characteristics of all participants and comparison of participants with and without a decrease in attention score on Day 1 after surgery. aaMAC, age-adjusted minimum alveolar concentration. [file Data_Sheet_1.docx]

**Supplementary Methods:**

### **EEG post-processing and data selection**

To remove noise and artifact, EEG data were post-processed in MATLAB (The MathWorks, Inc., Natick, MA, USA) as described using the EEGLAB toolbox (Delorme and Makeig 2004) and custom scripts. After recording, the raw EEG data were bandpass filtered using two hamming-windowed sinc FIR filters (high pass: 1 Hz half-amplitude cutoff and 1 Hz transition; low pass: 50 Hz half-amplitude cutoff and 20 Hz transition). The signal was then downsampled to 250 Hz and re-referenced to the common average. Next, the data were divided into contiguous epochs of non-overlapping 3-second windows; epochs with voltage artifacts > ±100 μV were excluded. Cardiac, electrocautery, and EMG artifacts were identified and removed via independent component analysis (ICA). An expert observer blinded to patient post-surgical outcomes examined raw EEG data for remaining artifacts. Channels with residual artifacts precluding analysis were interpolated using spherical spline interpolation (Perrin, Pernier et al. 1989) if adjacent channels had sufficiently clean data. Otherwise, channels with such artifacts were excluded. If more than one channel from the frontal ROI needed to be excluded, either pre-operatively or intra-operatively, the subject was excluded from further analysis due to insufficient data.

Pre-operative EEG was recorded in the awake state for 3 minutes with eyes closed. Correspondingly, we analyzed the first 3-minute section of EEG data during anesthesia/surgery that satisfied these seven criteria: 1) start time >5 minutes after incision; 2) end time >5 minutes before the end of surgery; 3) end-tidal nitrous oxide concentration < 1% throughout; 4) no major visible artifacts during the period; 5) no burst suppression during the period; 6) no ketamine, dexmedetomidine or lidocaine infusions started before or during the period; and 7) no bolus doses of ketamine or dexmedetomidine given in the preceding 60 minutes. These criteria were chosen to focus on the anesthetic plateau phase of the case (Ni, Cooter et al. 2019), and to avoid confounding effects of the other anesthetic adjunct drugs listed above, each of which has a different pattern of EEG spectral effects than do volatile anesthetics (Purdon, Pierce et al. 2013).

**Supplementary Table 1:** Intraoperative characteristics of all participants and comparison of participants with and without a decrease in attention score on Day 1 after surgery.

| **Variable** | **Overall Cohort**  **(N=50)** | **No Decrease in Attention Score**  **(N=32)** | **Decrease in Attention Score**  **(N=18)** | **P-value** |
| --- | --- | --- | --- | --- |
| **Surgery and Anesthetic Characteristics** | | | | |
| Surgery Service, n (%)  Thoracic Surgery  General Surgery  Gynecology  Orthopedics  Urology | 8 (16.0%)  11 (22.0%)  7 (14.0%)  9 (18.0%)  15 (30.0%) | 4 (12.5%)  8 (25.0%)  5 (15.6%)  7 (21.9%)  8 (25.0%) | 4 (22.2%)  3 (16.7%)  2 (11.1%)  2 (11.1%)  7 (38.9%) | 0.61^2^ |
| Anesthesia type, n (%)  General Anesthesia (GA)  GA + Regional/Epidural/Spinal  Spinal + MAC  Regional block + MAC  Spinal + Regional Block + MAC | 30 (60.0%)  17 (34.0%)  0 (0.0%)  1 (2.0%)  2 (4.0%) | 20 (62.5%)  9 (28.1%)  0 (0.0%)  1 (3.1%)  2 (6.3%) | 10 (55.6%)  8 (44.4%)  0 (0.0%)  0 (0.0%)  0 (0.0%) | 0.60^4^ |
| Surgery Duration in minutes, median [Q1, Q3] | 144 [110, 181] | 142 [115, 218] | 145 [104, 163] | 0.61^3^ |
| **Intraoperative Medications** | | | | |
| Propofol Dose (mcg/kg/min)* | 12.7 [8.6, 29.5] | 12.7 [8.5, 19.5] | 13.1 [8.7, 32.7] | 0.74^3^ |
| Paralytics Used **/**Dose (mg) | 41 (82.0%)/  80[65, 110] | 25 (78.1%)/  80 [70, 120] | 16 (88.9%)/  75 [63, 103] | 0.34^2^/  0.272^3^ |
| Ketamine Used / Dose (mcg/kg/min) | 9 (18.0%)/  3.0 [2.6, 3.4] | 6 (18.8%)/  3.1 [2.6, 4.5] | 3 (16.7%)/  3.0 [1.8, 3.3] | 0.85^2^/  0.57^3^ |
| Fentanyl or Hydromorphone Used / Dose (ME mg) | 41 (82.0%)/  15 [10, 25] | 25 (78.1%)/  15 [10, 25] | 16 (88.9%)/  13 [6, 26] | 0.34^2^/  0.67^3^ |
| Phenylephrine Used / Dose (mg) | 40 (80.0%)/  1.9 [0.3, 4.8] | 26 (81.3%)/  2.0 [0.3, 5.1] | 14 (77.8%)/  0.6 [0.1, 2.7] | 0.77^2^/  0.13^3^ |
| Midazolam Used / Dose (mg) | 7 (14.0%)/  2 [2, 2] | 5 (15.6%)/  2 [2, 2] | 2 (11.1%)/  2 [2, 2] | 0.66^2^/  0.75^3^ |
| Dexmedetomidine / Dose (mg) | 9 (18.0%)/  12 [8, 20] | 6 (18.8%)/  20 [8, 20] | 3 (16.7%)/  8 [8, 12] | 0.85^2^/  0.20^3^ |
| Epidural Used | 7 (14.0%) | 4 (12.5%) | 3 (16.7%) | 0.68^2^ |
| Inhaled Anesthetic used/ aaMAC hours | 46 (92%)/  0.32 [0.25, 0.46] | 29 (90.6%)/  0.3 [0.3, 0.4] | 17 (94.4%)/  0.4 [0.3, 0.5] | 0.63^2^/  0.54^3^ |
| N2O Used | 8 (16.0%) | 5 (15.6%) | 3 (16.7%) | 0.92^2^ |

aaMAC = age-adjusted minimum alveolar concentration

P-value key: 1=t-test, 2=Chi-Square, 3=Wilcoxon rank sum, 4=Fisher

**Supplemental Table 2: Comparison of participants with and without a crossover point in their pre-operative and intra-operative MSE curves**

|  | | | |
| --- | --- | --- | --- |
|  | Crossover Point Present (N=42) | Lack a Single Crossover Point (N=8) | Total (N=50) |
| **AGE** |  |  |  |
| Mean (SD) | 68.9 (5.5) | 68.0 (5.3) | 68.8 (5.4) |
| Range | (60-82) | (63-78) | (60-82) |
|  |  |  |  |
| **RACE** |  |  |  |
| Asian | 1 (2.4%) | 0 (0.0%) | 1 (2.0%) |
| Black or African American | 8 (19.0%) | 1 (12.5%) | 9 (18.0%) |
| Caucasian/White | 33 (78.6%) | 7 (87.5%) | 40 (80.0%) |
|  |  |  |  |
| **GENDER** |  |  |  |
| Female | 22 (52.4%) | 3 (37.5%) | 25 (50.0%) |
| Male | 20 (47.6%) | 5 (62.5%) | 25 (50.0%) |
|  |  |  |  |
| **Total MMSE score** |  |  |  |
| Mean (SD) | 27.48 (2.31) | 27.38 (3.58) | 27.46 (2.51) |
|  |  |  |  |
| **Self Rated Health** |  |  |  |
| 1 | 7 (16.7%) | 1 (12.5%) | 8 (16.0%) |
| 2 | 13 (31.0%) | 6 (75.0%) | 19 (38.0%) |
| 3 | 16 (38.1%) | 1 (12.5%) | 17 (34.0%) |
| 4 | 6 (14.3%) | 0 (0.0%) | 6 (12.0%) |
|  |  |  |  |
| **Depression Total Score** |  |  |  |
| Mean (SD) | 9.4 (8.1) | 12.1(6.0) | 9.8 (7.8) |
|  |  |  |  |
| **Instrumental Activities of Daily Living** |  |  |  |
| Mean (SD) | 6.7 (1.5) | 6.7 (1.4) | 6.7 (1.5) |
|  |  |  |  |
| **Body Mass Index** |  |  |  |
| Mean (SD) | 28.7 (5.5) | 32.0 (5.7) | 29.2 (5.6) |
|  |  |  |  |
| **Diabetes** | 5 (11.9%) | 1 (12.5%) | 6 (12.0%) |
| **Chronic Lung Disease** | 7 (16.7%) | 1 (12.5%) | 8 (16.0%) |
| **Cardiovascular Disease** | 11 (26.2%) | 1 (12.5%) | 12 (24.0%) |
| **Chronic Kidney Disease** | 5 (11.9%) | 0 (0.0%) | 5 (10.0%) |
| **Cerebrovascular Disease** | 1 (2.4%) | 0 (0.0%) | 1 (2.0%) |
| **Thyroid Disease** | 9 (21.4%) | 1 (12.5%) | 10 (20.0%) |
| **Rheumatoid Arthritis** | 1 (2.4%) | 0 (0.0%) | 1 (2.0%) |
| **SURGICAL_SERVICE** |  |  |  |
| Cardiothoracic | 7 (16.7%) | 1 (12.5%) | 8 (16.0%) |
| General Surgery | 10 (23.8%) | 1 (12.5%) | 11 (22.0%) |
| Gynecology | 6 (14.3%) | 1 (12.5%) | 7 (14.0%) |
| Orthopedics | 6 (14.3%) | 3 (37.5%) | 9 (18.0%) |
| Urology | 13 (31.0%) | 2 (25.0%) | 15 (30.0%) |
|  |  |  |  |
| **ANESTHESIA Type** |  |  |  |
| Missing | 14 (33.3%) | 0 (0.0%) | 14 (33.3%) |
| General with Epidural | 1 (3.6%) | 0 (0.0%) | 1 (2.8%) |
| General | 24 (85.7%) | 6 (75.0%) | 30 (83.3%) |
| General, Regional | 2 (7.1%) | 0 (0.0%) | 2 (5.6%) |
| Propofol Sedation, no Intubation | 0 (0.0%) | 2 (25.0%) | 2 (5.6%) |
| Regional | 1 (3.6%) | 0 (0.0%) | 1 (2.8%) |
|  |  |  |  |
| **Surgical Duration, Minutes** |  |  |  |
| Mean (SD) | 164.1 (94.5) | 136.9 (42.5) | 159.7 (88.5) |
|  |  |  |  |
|  | | | |
